# Chronological Change of Lumbar Symptoms After Anterior Cervical Discectomy and Fusion for Degenerative Cervical Radiculopathy at a Single Level

**DOI:** 10.7759/cureus.117876

**Published:** 2026-10-13

**Authors:** Yoshinori Maki, Hideki Hayashi, Toshinari Kawasaki, Akio Goda, Motohiro Takayama

**Affiliations:** 1 Department of Neurosurgery, Hikone Chuo Hospital, Hikone, JPN; 2 Department of Neurosurgery, Uji Tokushukai Hospital, Kyoto, JPN; 3 Department of Neurosurgery, Medical Research Institute Kitano Hospital, Public Interest Incorporated Foundation (PIIF) Tazuke-Kofukai, Osaka, JPN; 4 Department of Neurosurgery, Kyoto Katsura Hospital, Kyoto, JPN; 5 Department of Physical Therapy, Faculty of Health and Medical Sciences, Hokuriku University, Kanazawa, JPN

**Keywords:** anterior cervical discectomy and fusion, back pain evaluation questionnaire, cervical myelopathy evaluation questionnaire, degenerative cervical radiculopathy, japanese orthopaedic association score, lumbar symptoms, neck disability index, oswestry disability index, visual analog scale

## Abstract

Introduction: This exploratory retrospective study aimed to analyze the chronological impact on lumbar symptoms after anterior cervical discectomy and fusion (ACDF) for degenerative cervical radiculopathy (DCR).

Methods: Patients with DCR who underwent ACDF for a single level between January 2017 and September 2022 were included. The following scores from these questionnaires at postoperative year 1 were statistically compared: Japanese Orthopaedic Association (JOA) score, Back Pain Evaluation Questionnaire (BPEQ), Cervical Myelopathy Evaluation Questionnaire (CMEQ), Neck Disability Index (NDI), Oswestry Disability Index (ODI), and visual analog scale (VAS). Although statistical significance was defined as a p-value of <0.05, we did not interpret adjusted p-values confirmatively because this study is an exploratory analysis.

Results: A total of 29 patients (15 men and 14 women) with an average age of 60.8 years were enrolled in this exploratory study. Preoperative and postoperative cervical total JOA scores were 14.95±1.26 and 16.31±0.80, respectively. Preoperative CMEQ scores for cervical spine, upper extremities, lower extremities, bladder, and quality of life (QOL) were 64.66±22.36, 87.86±14.17, 88.79±12.26, 87.86±14.22, and 50.72±13.29, respectively. Postoperatively, those variables were 86.90±14.85, 95.10±8.08, 91.07±14.19, 89.79±14.55, and 59.38±15.12, respectively. Preoperative VAS scores for neck and shoulder, chest, upper extremities, and trunk/lower extremities were 64.28±26.72, 6.55±15.15, 60.86±30.18, and 12.93±20.53, respectively. Postoperatively, those scores were 45.86±33.75, 6.41±17.15, 41.55±35.70, and 22.33±30.03, respectively. Preoperative and postoperative NDI scores were 27.62±15.19 and 17.00±12.07, respectively. Significant improvements were identified in these variables. However, we did not identify any significant postoperative improvement in JOA III, CMEQ (lower extremities and bladder), VAS (chest and trunk/lower extremities), and lumbar questionnaire scores. A sub-analysis of patients with preoperative VAS (lumbar spine) of 20.00 or more, or ODI of 21.00% or more, did not show significant postoperative improvement in lumbar questionnaire scores, either.

Conclusion: The impact of ACDF at a single level for patients with DCR could be limited for preoperative lumbar symptoms.

## Introduction

A degenerative intervertebral disc and the formation of osteophytes can irritate the cervical nerve roots, resulting in degenerative cervical radiculopathy (DCR). This pathology results in radicular pain and motor weakness and affects the quality of life (QOL) of patients, and those symptoms can occur at a single level or multiple levels. Conservative management, including medication, a neck collar, and rehabilitation, is initially considered. When conservative management does not resolve the radicular symptoms of patients, surgical management can be considered [1,2].

Anterior cervical discectomy and fusion (ACDF) is an established surgical management for DCR, and symptoms related to cervical radiculopathy can be resolved after surgery when appropriate decompression is achieved [3-5].

As lumbar symptoms are a common health concern from the young to the elderly in daily practice [6,7], we encounter patients with DCR accompanied by preoperative lumbar symptoms. Postoperative amelioration or resolution of preoperative lumbar symptoms after surgery for degenerative cervical myelopathy (DCM) has been reported in several studies [8-12]. However, to our knowledge, no prior studies have focused on assessing the chronological change in lumbar symptoms after ACDF for patients with DCR. We hypothesized that ACDF in cases of DCR would not significantly affect preoperative lumbar symptoms, unlike DCM. Therefore, in this exploratory study, we evaluated the chronological impact of this surgical approach on coexisting preoperative lumbar symptoms.

## Materials and methods

Patients who underwent operations for cervical lesions between January 2017 and September 2022 were identified from our medical records at Kitano Hospital Medical Research Institute (n=263). Patients were not selected based on the presence or absence of preoperative lumbar symptoms; all consecutive patients who met the inclusion criteria were enrolled, and lumbar symptoms were assessed using the questionnaires described below. Patients enrolled in this study did not undergo lumbar magnetic resonance imaging (MRI) because of financial concerns. Clinical symptoms of patients were evaluated using the Japanese Orthopaedic Association (JOA) score [13], Back Pain Evaluation Questionnaire (BPEQ) [14], Cervical Myelopathy Evaluation Questionnaire (CMEQ) [15], Neck Disability Index (NDI) [16], Oswestry Disability Index (ODI) [17], and visual analog scale (VAS). The questionnaires were performed preoperatively and one year after ACDF. During the follow-up, no patients underwent lumbar surgery. We excluded patients who underwent cervical laminoplasty (n=213), those with insufficient clinical data (n=17), and those who underwent ACDF for ossification of the posterior longitudinal ligament (n=3) and for three levels (n=1). Finally, based on the preoperative lumbar questionnaire scores, we divided 29 patients into symptomatic (n=13) and minimally symptomatic lumbar (n=16) groups (Figure 1). Patients with preoperative VAS (lumbar spine) of 20.00 or more, or ODI of 21.00% or more, were categorized into the symptomatic group, while those without were in the minimally symptomatic group.

**Figure 1:**
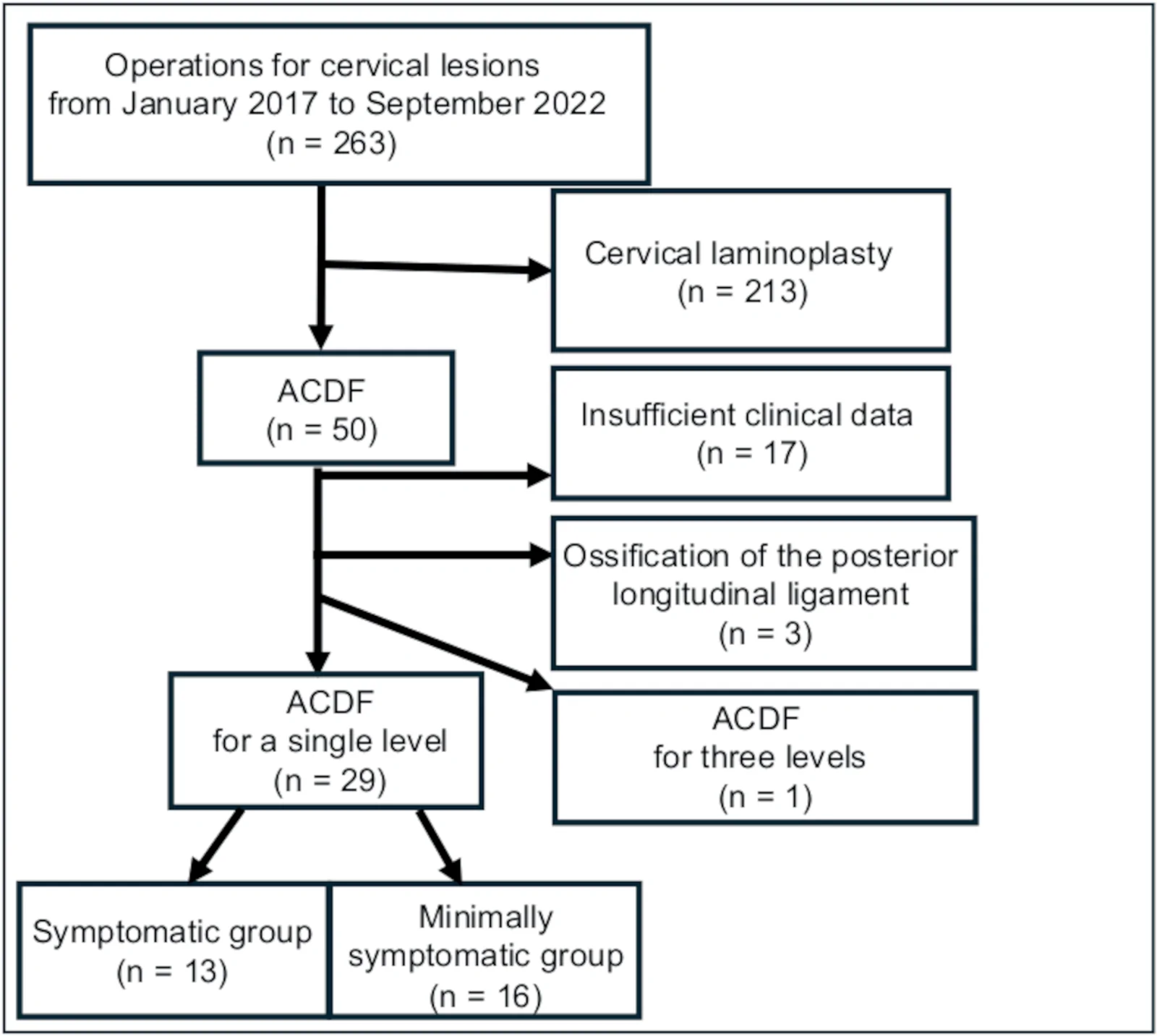
Flowchart of included and excluded cases in this study This flowchart shows the inclusion and exclusion criteria in this study. ACDF: anterior cervical discectomy and fusion

Surgical procedure

ACDF was indicated for cervical symptoms resistant to conservative management, such as medications and rehabilitation therapy. Patients were set in the supine position under general anesthesia. The upper body was slightly elevated. The approach side (right or left) was determined according to the surgeons' preference. The head was slightly rotated to the contralateral approach side. The target level was confirmed under C-arm fluoroscopy. A linear transverse skin incision at the targeted level was made. After cutting the platysma, we confirmed the anterior limb of the sternocleidomastoid muscle. Following the pathway medial to the carotid sheath and lateral to the trachea and esophagus, the connective tissue was bluntly dissected. The longus colli muscle was identified and detached from the vertebral bodies. The intervertebral disc was removed, and its space was replaced with an artificial intervertebral spacer. After confirming hemostasis, a drain tube was placed, and the wound was closed.

Statistical methods

Preoperative and postoperative scores were compared using the paired t-test when the distribution of the paired differences did not deviate from normality (Shapiro-Wilk test, p≥0.05), and the Wilcoxon signed-rank test otherwise. Holm and Benjamini-Hochberg adjustments were additionally applied within each family of 14 comparisons (cervical and lumbar). Effect sizes were calculated as r=|Z|/√n for the Wilcoxon signed-rank test and Cohen's dz for the paired t-test, and the Hodges-Lehmann estimator of the median difference with its distribution-free 95% confidence interval (CI) was reported. Spearman's rank correlation was used to examine the association between baseline scores and postoperative changes. Thereafter, to avoid the floor/ceiling effect of lumbar questionnaire scores, lumbar questionnaire scores were again evaluated in the symptomatic and minimally symptomatic lumbar groups. The results of the questionnaires were statistically analyzed using IBM SPSS Statistics software version 24 (IBM Corp., Armonk, NY), setting statistical significance at a p-value of <0.05. Because this study is an exploratory analysis, we did not interpret adjusted p-values confirmatively. This study was approved by the Kitano Hospital Medical Research Institute Ethics Committee (approval number: P2200501000).

## Results

This study enrolled 29 patients (15 men and 14 women). The average age (±standard deviation (SD)) was 60.8±10.9 years. Six patients underwent ACDF at C4/C5, 12 patients at C5/C6, 9 patients at C6/7, and 2 patients at C7/T1.

Preoperative and postoperative cervical total JOA scores were 14.95±1.26 and 16.31±0.80, respectively. The preoperative and postoperative subdivisions of the cervical JOA I, II, and III scores were 7.09±1.11, 4.90±0.57, and 2.97±0.19, and 7.72±0.69, 5.59±0.46, and 3.00±0.00, respectively. Preoperatively, CMEQ scores for cervical spine, upper extremities, lower extremities, bladder, and QOL were 64.66±22.36, 87.86±14.17, 88.79±12.26, 87.86±14.22, and 50.72±13.29, respectively. Meanwhile, postoperatively, those variables were 86.90±14.85, 95.10±8.08, 91.07±14.19, 89.79±14.55, and 59.38±15.12, respectively. Preoperatively, VAS scores for neck and shoulder, chest, upper extremities, and trunk/lower extremities were 64.28±26.72, 6.55±15.15, 60.86±30.18, and 12.93±20.53, respectively. Postoperatively, those scores were 45.86±33.75, 6.41±17.15, 41.55±35.70, and 22.33±30.03, respectively. Preoperative and postoperative NDI scores were 27.62±15.19 and 17.00±12.07, respectively. Significant improvements were identified in these variables, except for cervical JOA III, CMEQ (lower extremities and bladder), and VAS (chest and trunk/lower extremities). However, we did not identify any significant improvement between preoperative and postoperative lumbar questionnaire scores (Table 1).

**Table 1:** Pre- and postoperative clinical outcomes of the patients enrolled in this study (N=29) Analysis cohort: one procedure per patient, with complete preoperative and one-year data for both the cervical and the lumbar questionnaire sets. Wilcoxon signed-rank test for all comparisons except those marked * (paired t-test). Statistic = |Z| (tie-corrected) or |t| (df = 28). Multiplicity: Holm and Benjamini-Hochberg adjustment applied within each family of 14 comparisons (cervical, lumbar). Effect size: r = |Z| / sqrt(n) for Wilcoxon, Cohen dz for the paired t-test. Hodges-Lehmann = median of pairwise mean differences (POY1 minus preoperative) with a distribution-free 95% CI. "Baseline at best score (%)" = proportion of patients already at the most favorable possible value before surgery (100 for CMEQ/BPEQ, 0 for VAS/ODI); values ≥ 30% are shaded. VAS (chest): the test statistic is exactly 0.00 because the 10 non-zero differences produce identical positive and negative rank sums (27.5 each, with a tie at |31|). -: not applicable BPEQ: Back Pain Evaluation Questionnaire, CI: confidence interval, CMEQ: Cervical Myelopathy Evaluation Questionnaire, JOA: Japanese Orthopaedic Association, NDI: Neck Disability Index, ODI: Oswestry Disability Index, POY1: postoperative year 1, QOL: quality of life, SD: standard deviation, VAS: visual analog scale

	Preoperative					POY1					Test statistic	Unadjusted p-value	H-corrected p-value	BH-corrected q-value	Median difference	95% CI low	95% CI high	Effect size	Baseline at best score (%)
	Mean	SD	Median	Min	Max	Mean	SD	Median	Min	Max									
Cervical JOA_I	7.09	1.11	7.50	4.50	8.00	7.72	0.69	8.00	5.00	8.00	3.01	0.003	0.021	0.005	0.50	0.00	1.00	0.559	-
Cervical JOA_II	4.90	0.57	5.00	3.50	6.00	5.59	0.46	5.50	4.50	6.00	4.19	<0.001	0.000	<0.001	0.75	0.50	1.00	0.778	-
Cervical JOA_III	2.97	0.19	3.00	2.00	3.00	3.00	0.00	3.00	3.00	3.00	1.00	0.317	0.952	0.370	0.00	0.00	0.00	0.186	-
Cervical JOA	14.95	1.26	15.00	12.00	16.50	16.31	0.80	16.50	14.00	17.00	4.45	<0.001	0.000	<0.001	1.25	0.75	1.75	0.826	-
CMEQ (cervical spine)	64.66	22.36	60.00	20.00	100.00	86.90	14.85	95.00	65.00	100.00	3.95	<0.001	0.001	<0.001	20.00	12.50	30.00	0.733	17.2
CMEQ (upper extremities)	87.86	14.17	95.00	47.00	100.00	95.10	8.08	100.00	74.00	100.00	3.05	0.002	0.021	0.005	5.50	2.50	10.50	0.566	31.0
CMEQ (lower extremities)	88.79	12.26	95.00	64.00	100.00	91.07	14.19	100.00	50.00	100.00	1.19	0.235	0.941	0.299	0.50	-2.00	4.50	0.22	31.0
CMEQ (bladder)	87.86	14.22	94.00	44.00	100.00	89.79	14.55	94.00	38.00	100.00	0.65	0.516	1.000	0.556	0.00	-0.50	6.00	0.121	24.1
CMEQ QOL*	50.72	13.29	50.00	25.00	74.00	59.38	15.12	57.00	34.00	97.00	4.09	<0.001	0.003	0.001	8.50	3.50	13.50	0.76	-
VAS (neck and shoulder)	64.28	26.72	70.00	10.00	100.00	45.86	33.75	44.00	0.00	100.00	2.19	0.029	0.189	0.045	-19.50	-36.50	-3.00	0.406	0.0
VAS (chest)	6.55	15.15	0.00	0.00	53.00	6.41	17.15	0.00	0.00	70.00	0.00	1.000	1.000	1.000	0.00	0.00	1.00	0	75.9
VAS (upper extremities)	60.86	30.18	70.00	0.00	100.00	41.55	35.70	38.00	0.00	100.00	2.21	0.027	0.189	0.045	-18.50	-38.00	-3.00	0.411	3.4
VAS (trunk/lower extremities)	12.93	20.53	0.00	0.00	80.00	22.33	30.03	2.00	0.00	100.00	1.92	0.054	0.272	0.076	4.75	0.00	15.00	0.357	55.2
NDI (%)*	27.62	15.19	26.00	0.00	58.00	17.00	12.07	18.00	0.00	50.00	4.33	<0.001	0.002	0.001	-10.00	-16.00	-4.50	-0.803	-
Lumbar JOA_I	7.69	1.42	8.00	5.00	9.00	7.34	1.70	7.00	4.00	9.00	1.34	0.179	1.000	0.835	0.00	-0.50	0.00	0.25	-
Lumbar JOA_II	5.66	0.77	6.00	4.00	6.00	5.83	0.38	6.00	5.00	6.00	1.31	0.191	1.000	0.835	0.00	0.00	0.50	0.243	-
Lumbar JOA_III	11.45	2.18	12.00	7.00	14.00	11.48	2.85	12.00	4.00	14.00	0.25	0.806	1.000	0.873	0.00	-1.00	1.00	0.046	-
Lumbar JOA_IV	-0.93	1.98	0.00	-6.00	0.00	-0.41	1.05	0.00	-3.00	0.00	1.52	0.129	1.000	0.835	0.00	0.00	0.00	0.282	-
Lumbar JOA	23.86	4.41	24.00	12.00	29.00	24.24	4.86	25.00	11.00	29.00	0.53	0.593	1.000	0.873	0.50	-1.00	1.50	0.099	-
BPEQ (pain)	79.69	30.01	100.00	0.00	100.00	73.34	35.36	100.00	0.00	100.00	0.67	0.501	1.000	0.873	0.00	-14.00	0.00	0.125	58.6
BPEQ (lumbar spine)	84.69	22.80	100.00	8.00	100.00	85.07	22.90	100.00	17.00	100.00	0.40	0.686	1.000	0.873	0.00	-4.50	8.50	0.075	51.7
BPEQ (ambulation)	93.59	11.60	100.00	64.00	100.00	88.21	22.23	100.00	29.00	100.00	1.18	0.239	1.000	0.835	0.00	-10.50	0.00	0.219	65.5
BPEQ (social life)	74.24	21.07	73.00	14.00	100.00	77.66	22.21	78.00	19.00	100.00	0.24	0.810	1.000	0.873	3.50	-4.50	11.00	0.045	27.6
BPEQ (mental status)*	58.10	18.08	57.00	18.00	97.00	59.83	18.77	56.00	29.00	96.00	0.49	0.625	1.000	0.873	1.00	-5.50	7.50	0.092	0.0
VAS (lumbar spine)	22.07	27.06	10.00	0.00	86.00	26.07	27.63	15.00	0.00	95.00	0.76	0.445	1.000	0.873	2.50	-4.00	11.00	0.142	41.4
VAS (pain in the lower extremities)	10.38	18.47	0.00	0.00	75.00	14.93	25.63	0.00	0.00	80.00	0.78	0.437	1.000	0.873	0.00	-4.50	10.50	0.144	62.1
VAS (numbness in the lower extremities)	14.21	25.22	0.00	0.00	80.00	10.59	17.07	1.00	0.00	61.00	0.31	0.756	1.000	0.873	0.00	-2.00	3.50	0.058	62.1
ODI (%)	13.69	13.27	11.00	0.00	51.00	14.03	14.39	12.00	0.00	50.00	0.09	0.931	1.000	0.931	0.00	-3.00	2.00	0.016	31.0

Subgroup analysis

As a subgroup analysis, preoperative and postoperative lumbar questionnaires were compared in those patients with preoperative VAS (lumbar spine) of 20.00 or more, or ODI of 21.00 % or more. Although questionnaire scores improved postoperatively, these results were not statistically significant (Table 2).

**Table 2:** Lumbar outcomes in the symptomatic subgroup (n = 13) Subgroup threshold: preoperative VAS (lumbar spine) of 20.00 mm or more, or ODI of 21.00% (moderate disability) or more. Wilcoxon signed-rank test for all comparisons except those marked * (paired t-test). Spearman correlation between baseline score and change, whole cohort: VAS (lumbar spine) rho = -0.46 (p = 0.011); BPEQ (pain) rho = -0.41 (p = 0.027); BPEQ (lumbar spine) rho = -0.74 (p = 0.000); ODI (%) rho = -0.35 (p = 0.065), consistent with a floor/ceiling effect and regression to the mean. Every lumbar measure moves in the improving direction in the symptomatic subgroup. The subgroup is small; the analysis is exploratory, and the absence of significance should not be read as absence of effect. -: not applicable BPEQ: Back Pain Evaluation Questionnaire, CI: confidence interval, JOA: Japanese Orthopaedic Association, ODI: Oswestry Disability Index, POY1: postoperative year 1, SD: standard deviation, VAS: visual analog scale

	Preoperative					POY1					Test statistic	Unadjusted P-value	H corrected p-value	BH correction q-value	Median difference	95% CI low	95% CI high	Effect size	Baseline at best score (%)
	Mean	SD	Median	Min	Max	Mean	SD	Median	Min	Max									
Lumbar JOA_I	6.62	1.39	6.00	5.00	9.00	6.62	1.71	6.00	4.00	9.00	0.06	0.951	1.000	1.000	0.00	-1.00	1.00	0.017	-
Lumbar JOA_II	5.54	0.88	6.00	4.00	6.00	5.85	0.38	6.00	5.00	6.00	1.24	0.214	1.000	0.615	0.00	-0.50	1.00	0.345	-
Lumbar JOA_III	10.54	2.15	10.00	7.00	14.00	10.69	3.38	12.00	4.00	14.00	0.00	1.000	1.000	1.000	0.00	-2.00	2.50	0	-
Lumbar JOA_IV	-1.85	2.61	0.00	-6.00	0.00	-0.46	1.13	0.00	-3.00	0.00	1.86	0.063	0.823	0.443	1.50	0.00	3.00	0.515	-
Lumbar JOA	20.85	4.47	21.00	12.00	29.00	22.69	5.74	25.00	11.00	29.00	1.01	0.313	1.000	0.615	1.00	-2.00	6.50	0.28	-
BPEQ (pain)	61.31	33.70	71.00	0.00	100.00	65.85	34.76	71.00	0.00	100.00	0.85	0.395	1.000	0.615	0.00	-14.00	14.50	0.236	23.1
BPEQ (lumbar spine)	71.62	27.61	83.00	8.00	100.00	87.15	20.25	100.00	42.00	100.00	2.09	0.037	0.515	0.443	17.00	0.00	29.50	0.579	23.1
BPEQ (ambulation)	89.54	13.36	93.00	64.00	100.00	89.62	21.26	100.00	29.00	100.00	0.53	0.595	1.000	0.757	0.00	-3.50	14.50	0.147	46.2
BPEQ (social life)	67.77	22.55	70.00	14.00	100.00	79.15	25.13	86.00	19.00	100.00	1.12	0.262	1.000	0.615	11.00	-8.00	25.00	0.311	15.4
BPEQ (mental status)*	55.69	15.53	57.00	27.00	75.00	58.92	18.35	56.00	29.00	83.00	0.59	0.564	1.000	0.757	0.50	-8.00	12.00	0.165	0.0
VAS (lumbar spine)	45.15	24.93	47.00	0.00	86.00	37.23	27.44	44.00	0.00	90.00	1.07	0.284	1.000	0.615	-8.50	-24.00	9.00	0.297	7.7
VAS (pain in the lower extremities)	21.77	22.89	20.00	0.00	75.00	19.31	30.00	0.00	0.00	80.00	0.36	0.721	1.000	0.841	-4.50	-16.00	11.50	0.099	30.8
VAS (numbness in the lower extremities)	30.69	30.69	25.00	0.00	80.00	16.08	21.93	1.00	0.00	61.00	0.87	0.386	1.000	0.615	-5.00	-40.00	5.50	0.241	30.8
ODI (%)	24.92	10.40	26.00	9.00	51.00	18.77	14.51	20.00	0.00	50.00	1.57	0.116	1.000	0.541	-5.00	-13.00	0.50	0.436	0.0

The lumbar scores were deteriorated in the other subgroup analysis of patients with preoperatively minimal symptoms (Table 3).

**Table 3:** Minimally symptomatic subgroup (n = 16) Wilcoxon signed-rank test for all comparisons except those marked * (paired t-test). Every lumbar measure moves in the deteriorating direction in the minimally symptomatic subgroup. Spearman correlation between baseline score and change, whole cohort: VAS (lumbar spine) rho = -0.46 (p = 0.011); BPEQ (pain) rho = -0.41 (p = 0.027); BPEQ (lumbar spine) rho = -0.74 (p = 0.000); ODI (%) rho = -0.35 (p = 0.065), consistent with a floor/ceiling effect and regression to the mean. The subgroup is small; the analysis is exploratory, and the absence of significance should not be read as an absence of effect. -: not applicable BPEQ: Back Pain Evaluation Questionnaire, CI: confidence interval, JOA: Japanese Orthopaedic Association, ODI: Oswestry Disability Index, POY1: postoperative year 1, SD: standard deviation, VAS: visual analog scale

	Preoperative					POY1					Test statistic	Unadjusted P-value	H corrected p-value	BH correction q-value	Median difference	95% CI low	95% CI high	Effect size	Baseline at best score (%)
	Mean	SD	Median	Min	Max	Mean	SD	Median	Min	Max									
Lumbar JOA_I	8.56	0.63	9.00	7.00	9.00	7.94	1.48	9.00	4.00	9.00	1.93	0.054	0.644	0.158	-0.50	-1.00	0.00	0.482	-
Lumbar JOA_II	5.75	0.68	6.00	4.00	6.00	5.81	0.40	6.00	5.00	6.00	0.41	0.679	1.000	0.792	0.00	-0.50	0.50	0.104	-
Lumbar JOA_III	12.19	1.97	13.00	9.00	14.00	12.13	2.25	13.00	7.00	14.00	0.54	0.589	1.000	0.750	0.50	-1.00	1.00	0.135	-
Lumbar JOA_IV	-0.19	0.75	0.00	-3.00	0.00	-0.38	1.02	0.00	-3.00	0.00	1.00	0.317	1.000	0.494	0.00	0.00	0.00	0.25	-
Lumbar JOA	26.31	2.44	26.50	22.00	29.00	25.50	3.74	26.50	17.00	29.00	0.00	1.000	1.000	1.000	0.00	-3.00	1.00	0	-
BPEQ (pain)	94.63	15.55	100.00	43.00	100.00	79.44	35.75	100.00	0.00	100.00	1.48	0.140	0.980	0.245	0.00	-43.00	0.00	0.369	87.5
BPEQ (lumbar spine)	95.31	9.59	100.00	67.00	100.00	83.38	25.38	100.00	17.00	100.00	1.61	0.107	0.855	0.214	-4.25	-29.00	0.00	0.403	75.0
BPEQ (ambulation)	96.88	9.08	100.00	64.00	100.00	87.06	23.61	100.00	43.00	100.00	1.83	0.068	0.693	0.158	0.00	-25.00	0.00	0.456	81.3
BPEQ (social life)	79.50	18.86	78.00	43.00	100.00	76.44	20.31	78.00	38.00	100.00	0.77	0.441	1.000	0.617	0.00	-15.00	10.50	0.193	37.5
BPEQ (mental status)*	60.06	20.20	59.50	18.00	97.00	60.56	19.67	55.00	36.00	96.00	0.11	0.916	1.000	0.986	0.25	-8.00	10.50	0.027	0.0
VAS (lumbar spine)	3.31	5.69	0.00	0.00	18.00	17.00	25.04	7.00	0.00	95.00	2.40	0.016	0.228	0.158	6.00	0.00	23.00	0.601	68.8
VAS (pain in the lower extremities)	1.13	3.77	0.00	0.00	15.00	11.38	21.82	0.00	0.00	65.00	1.86	0.063	0.693	0.158	2.50	0.00	24.50	0.465	87.5
VAS (numbness in the lower extremities)	0.81	2.56	0.00	0.00	10.00	6.13	10.57	1.50	0.00	37.00	2.21	0.027	0.349	0.158	2.50	0.00	13.00	0.553	87.5
ODI (%)	4.56	6.65	0.00	0.00	18.00	10.19	13.53	3.00	0.00	40.00	1.84	0.066	0.693	0.158	2.00	0.00	13.50	0.46	56.3

## Discussion

In this exploratory study, we investigated the chronological change in lumbar symptoms after ACDF at the single-level DCR. Although we did not interpret p-values confirmatively, DCR symptoms improved after surgery, as shown by the JOA, CMEQ, and VAS scores. These findings seem to correspond to previous studies in which the effectiveness of ACDF for radiculopathy was proven [18]. However, concerning the lumbar symptoms evaluated with lumbar JOA, BPEQ, VAS, and ODI, there were no significant improvements. Although we did not identify any clinical contribution of ACDF for DCR for lumbar symptoms, this study is unique in that cervical and lumbar symptoms were simultaneously assessed in patients with DCR who underwent ACDF at a single level.

Unlike patients with DCR, postoperative improvement in lumbar symptoms in patients with DCM was described [8-12]. Several hypotheses have been postulated regarding the nascent mechanism of lumbar symptoms related to DCM, such as compression of the spinothalamic tract in the cervical regions, central sensitization accompanied by a triggered pain response in the lumbar regions, arterial and/or venous obstructions, ischemia of the thoracic cord, sagittal spinal malalignment as an adaptive response to DCM, psychological factors, and decreased physical activity and exercise caused by cervical sensory disturbance [10,12]. The decreased physical performance could have also resulted in preoperative lumbar symptoms in patients with DCR. Psychological factors might be causative of lumbar symptoms; however, there was no significant improvement in BPEQ (mental status) in our data. As the impact of ACDF on postoperative lumbar lordosis change is considered limited, it seems coherent that preoperative lumbar symptoms remained even after surgery [19]. The limited number of patients enrolled in this study could have also affected the results. Because we did not assess radiological and physiological variables simultaneously combined with cervical and lumbar questionnaires in our study, this topic warrants further research.

Interestingly, significant improvements were not observed in the scores of the Cervical JOA III (bladder function), CMEQ (lower extremities and bladder), and VAS (chest and trunk/lower extremities) in our study. Given the pathology of DCR, degenerative structures usually do not irritate the spinal cord. Therefore, ACDF is not effective for symptoms in the bladder, trunk, and lower extremities, which may result from DCM [20,21].

Regarding lumbar questionnaires, mean VAS scores in the lumbar spine and pain in the lower extremities were aggravated from 22.07 to 26.07 and from 10.38 to 14.93, respectively. Because our study included both patients with apparent lumbar symptoms (Table 2) and those with slight symptoms and full scores in BPEQ and VAS (Table 3), a floor effect and regression to the mean could affect the lumbar questionnaire scores. The mean JOA, BPEQ, VAS, and ODI scores improved in patients with preoperatively apparent lumbar symptoms (Table 2). Meanwhile, in patients with slight lumbar symptoms, those questionnaire scores were aggravated after surgery. These findings seem to be also related to the limited impact of ACDF on the lumbar lordosis [19], chronological lumbar degeneration, or emerging lumbar symptoms after disappearance of cervical symptoms. However, as there was no statistical significance in lumbar questionnaires, the effect of ACDF for patients with preoperative lumbar symptoms may still be limited. Therefore, clinicians should obtain informed consent from patients with DCR who will undergo ACDF, as some patients may also expect improvement in preoperative lumbar symptoms.

Limitations

This is an exploratory retrospective study that enrolled a limited number of patients. Especially, the number of patients in the sub-analysis group was very small. The follow-up period was relatively limited. Further research, including numerous patients followed over a long-term period, should be warranted. Multiple surgeons performed ACDF, and the surgical fashion was not completely standardized. Therefore, a performance bias might not have been completely excluded. As for the choice of surgical approach, posterior decompression was mostly performed. This is possibly because of the prevalence of ossification of the posterior longitudinal ligament and developmental cervical canal stenosis in Japan. Besides, we did not evaluate the correlation between clinical symptoms and radiological/physiological findings. Especially, we did not perform lumbar MRI examinations to rule out a tandem stenosis lesion. Although we used clinical questionnaires, such as the lumbar JOA, the subjective lumbar symptoms might not have been completely evaluated. This is possibly because preoperative JOA scores in some patients were full, and this can be a floor effect. Lastly, we did not compare patients who underwent ACDF with those who were managed only with conservative treatment. This should also be addressed in other studies.

## Conclusions

In this study, we assessed the chronological change in preoperative lumbar symptoms after ACDF for DCR at a single level. Postoperative improvement in preoperative lumbar symptoms was not significant in our cohort. This is probably because patients without apparent preoperative lumbar symptoms were enrolled simultaneously in our study. Although lumbar questionnaire scores improved in patients with preoperative lumbar symptoms, because of a lack of statistical power, this finding was not sufficient to support the effectiveness of ACDF for preoperative lumbar symptoms. Considering the surgical indication for ACDF for DCR, this surgery might not be effective for preoperative lumbar symptoms. Some patients may overinterpret surgical effectiveness; therefore, prudent informed consent seems warranted. To rule out other pathologies causing preoperative lumbar symptoms, additional examination and management should be considered. To clarify the effectiveness of ACDF for DCR against preoperative lumbar symptoms, further research enrolling numerous patients seems warranted.
